# Chronically ill Canadians’ experiences of being unattached to a family doctor: a qualitative study of marginalized patients in British Columbia

**DOI:** 10.1186/1471-2296-13-69

**Published:** 2012-07-16

**Authors:** Valorie A Crooks, Gina Agarwal, Angela Harrison

**Affiliations:** 1 Department of Geography, Simon Fraser University, 8888 University Dr, Burnaby, BC, V5A 1 S6, Canada; 2 Department of Family Medicine, McMaster University, 1280 Main Street West, Hamilton, ON, L8S 4 L8, Canada

## Abstract

**Background:**

Unattached patients do not have a regular primary care provider. Initiatives are being developed to increase attachment rates across Canada. Most existing attention paid to patient unattachment has focused on quantifying the problem and health system costs. Our purpose is to qualitatively identify the implications of chronically ill patients’ experiences of unattachment for health policy and planning to provide policy-relevant insights for Canadian attachment initiatives.

**Methods:**

Three focus groups were conducted with marginalized chronically ill individuals residing in a mid-sized city in British Columbia who are unattached to a family doctor. We use the term marginalized as a descriptor to acknowledge that by virtue of their low socio-economic status and lack of attachment the participants are marginalized in Canada’s health care system Focus groups were structured as an open conversation organized around a series of probing questions. They were digitally recorded and transcribed verbatim. Thematic analysis was employed.

**Results:**

Twenty-six individuals participated in the focus groups. The most common chronic illnesses reported were active drug addiction or recovery (and their associated symptoms), depression, arthritis, and hepatitis C. Participants identified life transitions as being the root cause for not having a family doctor. There was a strong sense that unsuccessful attempts to get a family doctor reflected that they were undesirable patients. Participants wanted to experience having a trusting relationship with a regular family doctor as they believed it would encourage greater honesty and transparency. One of the main health concerns regarding lack of access to a regular family doctor is that participants lacked access to preventative care. Participants were also concerned about having a discontinuous medical record due to unattachment.

**Conclusions:**

Participants perceived that there are many benefits to be had by having attachment to a regular family doctor and that experiencing unattachment challenged their health and access to health care. We encourage more research to be done on the lived experience of unattachment in order to provide on-the-ground insights that policy-makers require in order to develop responsive, patient-centred supports and programs.

## Background

Unattached patients do not have a regular primary care provider and are often left to seek basic and preventative health care through walk-in clinics and emergency rooms [1,2]. It has been well established that there is a significant number of unattached patients in Canada [2], with some media reports estimating that up to five million Canadians do not have a regular family doctor [3,4]. A recent estimate suggests that there are 3200 fewer family doctors practicing in Canada than what is needed to meet demand [5]. A 2008 survey reported that approximately 25% of Canadians experience some form of barrier to accessing regular medical care [6] and a 2009 survey found that 13% experience barriers to routine or ongoing primary care specifically [7]. Given the structure of Canada’s public health care system, the existence of unattached patients is problematic. This is because Canadian family doctors serve as ‘gatekeepers’ to secondary and tertiary care, whereby referral from a family doctor is required for a patient to see a specialist or obtain surgery [8-10]. This aspect of the organization of the public health care system heightens the need for Canadian patients to have a regular family doctor above and beyond the preventative care and treatment they offer to patients [11].

It is thought that there are a number of reasons as to why Canadian patients become and/or remain unattached to a family doctor, including the: closure of rural practices, steady stream of new immigrants, and health care planning mandates that have not accurately anticipated physician demand [12-14]. In response, a number of regional and provincial/territorial health administration bodies have created initiatives in the form of policies and programs designed to increase the numbers of practicing family doctors [15]. These initiatives exist typically at regional and provincial/territorial levels because while the Canadian public health care system is partially funded federally, it is administered on a provincial or territorial basis [16]. For example, in the province of British Columbia (BC), the BC Ministry of Health has developed the *Attachment Initiative*, which is designed to ensure that all residents of the province who want a family doctor have access to one [17,18]. Among other features, the program encourages family doctors to take new high-needs patients into their practice, including chronically ill individuals and seniors [17,19]. Initiatives such as this one have been implemented across the country [20-22].

Existing studies have identified the health risks that patients face as a result of being unattached to a family doctor. For example, such patients are less likely to receive preventative care, such as routine blood pressure checks [23-25]. Conversely, studies have also demonstrated the benefits of attachment. Among these is that having a regular family doctor increases the likelihood of having an annual medical visit [25,26]. Attached patients have also stressed the importance of being able to have a continuous medical record and the benefits of seeing a family doctor who is familiar with their family health history [27-29]**.**

Patients can have a regular place of care without having attachment to a regular provider [30,31]. This is true for those who are reliant on walk-in clinics. In Canada, walk-in clinics prioritize offering care without an appointment [32]. The medical care provided in these clinics is funded by the public system, and thus does not require out-of-pocket payment. While some walk-in clinics allow patients to request a particular physician, this varies by site and is often not allowed because the walk-in clinic model is built around interpersonal discontinuity [33]. These clinics are commonly visited by patients who have a regular family physician but need care for a non-emergency health issue for which they do not want to wait to see their regular doctor [33]. There are some patients, however, for whom the walk-in clinic is their regular place of care [33]. Although there are benefits to be had from having a regular place of care, such as having a continuous medical record being kept [34], in a health care system predicated on gatekeeping at the primary care tier in order to access specialized care like Canada’s, having a regular care site alone poses as a barrier to obtaining referrals due to a lack of continuous and ongoing monitoring by a single knowledgeable physician [32].

In order to inform initiatives aiming to address the problem of unattached patients in Canada, it is imperative that consideration be given to the experiential accounts of unattached patients. This is because knowledge end-users place increasing value on the role that personal accounts and qualitative evidence more broadly can play in decision-making processes [35-37]. Meanwhile, most existing attention paid to unattachment has focused on quantifying the problem and health system costs [1,12,25,38,39]. To address this knowledge gap, in the present article we examine the experiences of unattached patients in a low socio-economic status neighbourhood of a mid-sized BC city who are managing chronic illnesses in order to provide policy-relevant qualitative insights. By virtue of their low socio-economic status and lack of attachment they are marginalized in Canada’s health care system. We focus on chronic illnesses because people managing chronic illnesses require timely access to care [40] and yet are more likely to be unattached than those without chronic health conditions [12]. This may be due, in part, to the fact that Canadian family doctors can choose who to accept into their practices, and so it is thought that difficult or challenging patients are screened out [20].

## Methods

We ran focus groups [41,42] with chronically ill individuals residing in a low socio-economic status neighbourhood of a mid-sized BC city who self-identified as being unattached to a family doctor. The city was selected as there had been local media coverage of a doctor shortage coupled with challenges recruiting new family doctors [43]. There are nine walk-in clinics, 6 family medicine practices that offer some walk-in services (which are sometimes limited to patients enlisted in the practice), and over 100 family doctors (not all of whom have full-time hours or are in regular practice) in the city. We focused on a low socio-economic neighbourhood status as we wanted to speak with people would not likely have the means to travel outside the city in order to seek attachment to a non-local family doctor.

### Recruitment

We sought to have 5 to 10 participants at each of three focus groups. To recruit participants, a research assistant posted study advertisements in local shops and community centres. Those interested in participating were asked to contact the research assistant directly. At that point, potential participants were asked a series of questions in order to determine their eligibility. Our inclusion criteria were that participants self-identified as being: (1) over the age of 18; (2) chronically ill; (3) unattached to a family doctor; and (4) a resident of the city. Those who met these criteria were booked into one of the three focus group times, based upon preference. Prior to undertaking recruitment, we first applied for and received ethical approval for the study from the Office of Research Ethics at Simon Fraser University.

### Data collection

All focus groups were held at a local coffee shop that had community meeting rooms. Refreshments were provided throughout, and each participant was also given a CND$30 honorarium upon completion. At the start of each group participants were given a consent form to sign and return as well as a demographic questionnaire consisting of 16 questions. The purpose of the questionnaire was to gather background information on the participants.

All focus groups were facilitated by the first author, while a research assistant served as note taker. All groups ran for two hours each. To start, the facilitator and note taker introduced themselves, after which an overview of the focus group goals and ground rules (e.g., no interrupting) were provided. Following this, each person was asked to take a moment to explain why it was they did not have a regular family doctor. The remainder of the focus group was an open conversation organized around probing questions that were developed based on our review of the unattached patient literature: (1) what is important about having a regular family doctor; (2) what do you want to be able to visit with a family doctor about; (3) how do you deal with your health concerns without having a family doctor; (4) how do you go about seeing a doctor now; (5) what specific concerns do you have about not having a regular family doctor; (6) how do you feel about not having a regular family doctor; and (7) what do you think you need to do in order to obtain a family doctor. The open conversation portion of each of the focus groups flowed naturally and was highly interactive, and so little facilitation was required.

### Data analysis

The focus groups were digitally recorded and verbatim transcriptions were made. Thematic analysis was used to examine the dataset. Thematic analysis involves categorizing qualitative data into themes that are based upon patterns within a dataset and refined when compared to the literature [44]. We first independently read the transcripts to identify emerging themes. We then held a series of meetings in order to discuss these themes and compare them to what we had learned from the literature. Next, two external readers – i.e., people who were not involved with proposing or running the study – with established qualitative data analysis skills reviewed the anonymized transcripts for to confirm the dominance of the identified and our interpretation of their parameters [45]. This added to the rigour of our process [46].

Following theme identification and confirmation, the transcripts were reviewed again by all investigators in order to build an interpretive matrix, where the cells were populated with summary points of the findings unique to each theme and relationships between themes were established [47]. Following discussion of the parameters of each theme, the third author culled from the dataset extracts from the transcripts that related to each and stored them in separate files as a way of coding and organizing the data. This coding process was undertaken in a word processing program given the small size of the dataset. A second investigator reviewed these extracts to be sure that they had been assigned to the appropriate theme. A final meeting was then held in order review the coded data, confirm once again the parameters of the themes, identify links between the themes, and select quotes that best represent the sub-themes to be used in the current article.

## Results

Twenty-six individuals ranging in age from 20 to 58 years of age (average = 37) – thirteen men and thirteen women – participated in the focus groups. All participants self-identified as having at least one chronic illness. Nine reported having one, seven reported having two, while ten had three or more chronic illnesses. The chronic illnesses reported were: active addition or addiction recovery, including the associated physical symptoms of both (n = 16); arthritis or other rheumatic disease (n = 8); depression (n = 7); hepatitis C (n = 5); attention deficit hyperactivity disorder (n = 4); high blood pressure (n = 2); diabetes (n = 1); hypoglycemia (n = 1); Crohn’s disease (n = 1); liver disease (n = 1); and post-traumatic stress disorder (n = 1). Two participants reported also having very rare chronic illnesses that we do not mention here by name to protect anonymity, and a third chose not to disclose the illnesses he was managing. All but two were receiving some form of government income assistance at the time of data collection. They had lived in the community of focus anywhere between two weeks and 38 years, with the average being just over four years.

All participants self-identified as being unattached to a family doctor, though some did have a regular place of care (i.e., a usual walk-in clinic). Most participants identified life transitions (e.g., moving, release from prison, living in transitional housing) as being the root cause of their lack of attachment. In the remainder of section we examine the four dominant themes that contribute to understanding participants’ lived experiences of unattachment: (1) coping with unattachment; (2) accessing preventative care; (3) health record and administration challenges; and (4) perceived interpersonal benefits of attachment. Direct quotations are provided to convey participants’ experiential accounts, noting in brackets the focus group number.

### Coping with unattachment

One way that some participants coped with not having a regular family doctor was to use walk-in clinics. Sixteen participants cited reliance on walk-in clinics, with some going to these clinics more than once per month and others using them infrequently. The ten participants who did not use walk-in clincis either reported having no ongoing strategy for accessing care or avoiding health services altogether by “sucking it up” (FG2). While some of the16 who used walk-in clinics visited a single clinic regularly, others had no clinic preference and visited whichever one was closest or had the shortest wait time when they were in need of care. Using walk-in clinics was collectively seen by the participants as the best way to address their health needs while being unattached, although there were some notable limitations:

"But unfortunately, when you just see a rotating doctor…I think it’s just, the matter of fact, is that I should just probably be more aware of my health, and seek out someone more competent for certain things that I’ve got going on with me, right. (FG1)"

A minority of participants reliant on walk-in clinics were very satisfied with this strategy of coping with unattachment, reporting that “they’re good doctors” (FG3). Even in cases where participants were satisfied with the care received, there was still a desire to attain attachment to a regular family doctor.

A problem-based coping strategy participants employed to manage unattachment was to undertake active measures to get onto the roster of a family doctor. These measures included asking others if they knew of family doctors accepting patients, seeking assistance from counsellors, and looking for information online. These active measures had, however, not been successful. One reason for this lack of success is that it was thought that some family doctors might not want to accept new patients because they want to keep their client loads low. It was also explained that family doctors can “choose who their patients will be” (FG3), and it was thought that chronically ill patients, and particularly those managing addictions, were undesirable to take on because they require ongoing care and attention. One participant went as far as to say that: “…they’re [family doctors] discriminating against us” (FG1). Although participants did not like the fact that doctors had some choice over which patients they took on, there was some understanding of why this might take place: “It’s not fair, but if I were a doctor, I’d pick and choose. I’d want people that were not going to give me trouble…” (FG3). In general, there was a strong sense that participants’ unsuccessful attempts to gain attachment meant that they were undesirable patients. This was thought to be most clearly demonstrated in cases where participants had been interviewed by family doctors who were accepting new patients only to ultimately not be taken onto their rosters. Part of managing unattachment thus involved coping with the notion that one might be an undesirable patient in addition to seeking out ways to become attached.

### Accessing preventative care

Although participants reported experiencing challenges obtaining referrals to secondary and tertiary care providers because of not having a regular family doctor, what they were most concerned about was lack of access to ongoing or preventative care at the primary tier. Instead of visiting a family doctor on a regular basis for chronic illness management and scheduled check-ups, as one individual pointed out, “if you go to a walk-in clinic, you only…walk in when you feel sick, or when you think you’re sick” (FG1). Participants explained that they typically only visited walk-in clinics when symptoms were noticeably exacerbated or when it was apparent that they were dealing with a new condition or symptom, never going for preventative care. This left most participants with waiting until ‘crisis point’ with their chronic illnesses until seeking medical attention, which left some visiting the emergency room more often than they would like. As one participant told:

"…I don’t have a family doctor, and I don’t want to go sit and wait in a clinic…so I’m just going to let it [new health condition] slide… But it’s [health] getting worse and worse, and it comes to the point where I can barely even eat anything, so I had to go to the emergency… (FG2)"

The above example, as well as other experiences described by participants, pointed to the fact that without a family doctor they tended to only seek medical help when they knew they were unwell, rather than as a form of preventative care to help keep them well. They perceived that they would have better access to preventative care and a stronger desire to obtain it through having attachment to a regular family doctor.

Because the participants lacked a regular family doctor, they were concerned that they had no person to prompt them with reminders about annual physical exams, blood work, or vaccinations. Instead, participants had to possess the diligence and desire to monitor and schedule preventative care or routine monitoring for themselves, which can be imperative for certain chronic conditions. Meanwhile, the absence of a family doctor made scheduling regular monitoring tests difficult:

"And it’s frustrating, because…you can’t get anything done. I guess, like I suffer from [hepatitis C], and…like, I’m supposed to have consistent blood tests done, to see where my levels are at, and I can’t, because I haven’t got a regular doctor. There’s no one to monitor them, right? (FG1)"

The health risks are clear in cases such as this one and others where people with conditions that require regular monitoring cannot readily access referrals for blood work. Many participants experienced such risks as a part of their everyday lives as a result of unattachment.

### Health record and administration challenges

A challenge participants experienced as a result of both unattachment and life transitions was that they had discontinuous or scattered medical records. While many acknowledged that their own moves and transitions had contributed to this situation, they also believed that if they had a regular family doctor s/he would oversee compiling their record through requesting transfers from clinics they had been treated at over time. Participants thought that an onus was placed upon them to remember important medical information, including recalling details of procedure dates and prescriptions:

"I injured my back…and if I have any future back injuries…they’re not going to know what disc, because I don’t remember which one I put out… But if the doctor doesn’t write in my file, “This is what he’s done. I prescribed this, he… and this is the kind of physio he took,” they’re not going to know any of that, right? (FG3)"

It was also suggested that an incomplete medical record may lead to wasting time and money when tests are repeated because previous results are not available. Concern was also expressed over the fact that prescription histories were discontinuous. As one participant recalled, “…I needed puffers [inhalers]. And I’m diabetic, and when they [walk-in clinic] gave me the prednisone, nobody told me not to take my puffer… So I ended up almost in a diabetic coma” (FG2).

Issues pertaining to health-related administration such as difficulties with completing paperwork and obtaining specialist referrals were raised on multiple occasions as challenges participants faced due to unattachment. For example, in order to receive disability benefits through social assistance, certain paperwork must be filled out by a doctor. It was explained that this presented a challenge for unattached patients. A participant shared his experience: “I tried to get [disability benefits]. I walked into the walk-in clinic…and I tried to get them to fill out my paperwork. He [the doctor] refused [due to a lack of familiarity with his case]” (FG1). Others experienced similar challenges in having paperwork completed for disability benefits, workplace programs, rehabilitation programs, and other forms of assistance. Interestingly, it was also noted several times that a regular family doctor might choose to waive the fee regularly associated with signing a patient’s paperwork due to their familiarity with a patient and his/her financial status. This was seen as a related benefit of attachment.

### Perceived interpersonal benefits of attachment

Participants reflected not only on what the practical benefits of having attachment to a family doctor could be (e.g., preventative care reminders, continuous medical record), but also the interpersonal benefits of developing a strong, trusting doctor-patient relationship. Participants wanted to experience having a trusting relationship with a regular family doctor as they believed that it would encourage greater honesty and transparency on their part:

"Like, if you go to see a different person [doctor] every time, there’s no level of trust there. So you’re not really going to get the help you need if you’re not really opening up and telling them what’s really going on. (FG1)"

"Or to even open up, when you’re going to strange doctors, to be able to open up when there’s really a problem. You know, you might not be willing to do that, or say what you would if you had built trust in a family doctor. (FG3)"

Experiencing a trusting relationship was also thought to increase comfort during care because, “they know you, and when it’s personal things… [I] kind of want a family doctor for that” (FG2). The development of a trusting relationship was also thought to be an indicator of a patient no longer being “…another number in the system” (FG1). Participants thought that being and feeling known would heighten the levels of honesty, transparency, and comfort that could ultimately be achieved in a relationship with a regular family doctor.

Avoiding medical care or relying walk-in clinics made it difficult for participants to achieve any form of continuity of care. For example, it was explained that doctors at a walk-in clinic, “…don’t see the deterioration. They just see what you’re walking in with, the symptom you have at present. They’re not seeing that, you know, you’ve gone from being completely healthy to being completely sick…” (FG3). Beyond observing changes in health status over time, it was also suggested repeatedly that having attachment would lessen patients’ abilities to be deceptive because through forming a trusting relationship a regular doctor would come to know the patient and would diffuse any attempts at manipulation. As one participant explained, “if you see the same [doctor] all the time, they’ll learn something about you, enough to know when you’re [lying to] them and when you’re not…to see if you’re trying to just get drugs…” (FG1). Meanwhile, it was noted that patients could easily manipulate the walk-in clinic system in order to obtain desired, but not necessarily needed, pharmaceuticals or medical care.

## Discussion

The results show that the 26 participants perceive that there are a number of benefits of having a regular family doctor, and that because they lack such attachment they face certain negative consequences for their health and access to health care. In this section we consider the contributions of these results to our understanding of unattachment and the implications for Canadian health policy and planning. We also give consideration the limitations and strengths of the study.

### Insights on unattachment

In comparing the findings of this study to the existing literature, it can be understood that there are expected and unexpected reasons for why participants want to have attachment to a regular family doctor. Studies commonly find that unattached patients are less likely to receive preventative care through routine check-ups and screenings when compared to attached patients [23-25]. It was thus not surprising that this issue was raised by the participants, many of whom viewed having access to preventative care as incentive for becoming attached. However, added complexity comes from the fact that they are managing chronic illnesses that sometimes require regular monitoring through blood tests and other measures. Participants’ risk of experiencing negative health outcomes due to unattachment could be heightened when compared to unattached patients not managing chronic illnesses due to lack of access to measures that assist with ongoing monitoring and management. Further research is required to demonstrate whether or not this is occurring.

Having informational continuity of care through the establishment and upkeep of a solitary medical record is an expected reason why unattached patients to want to have a family doctor, and this was true for the focus group participants. Continuity of care research has demonstrated the health benefits of having informational continuity and that chronically ill patients in particular have much to gain from having a regular and continuous medical record [48-50]. The participants implicitly understood these benefits, and thus it was not surprising that concerns regarding having a discontinuous and incomplete medical record came up in our discussions about unattachment.

While it was not surprising to learn that participants implicitly valued the potentially trusting relationship that could be established with a family doctor, it was unexpected to learn of how they expected this to extend to a regular doctor identifying and monitoring manipulative or deceptive behaviours. This was a particular concern among participants with active drug addictions or in recovery. The establishment of a trusting relationship between doctor and patient is at the core of family medicine practice [51-53]. Certainly, this trust is something that develops over time through repeated visits and open communication [54-56]. The focus group participants viewed family doctors’ oversight of patients’ manipulative and deceptive behaviours to be a natural extension of this trusting relationship, seeing it as germane to the relationship itself. Their perception that this is how a trusting relationship with a family doctor could be experienced can be interpreted in several ways. One is that they see that having a continuous relationship with a single family doctor as heightening their own accountability. A second is that they want to shift the consequences of successfully manipulating a doctor onto the regular family doctor for not having identified it and off of the patient who initiated it. These and other interpretations need to be confirmed or refuted through further research prior to acting on this finding.

### Implications for Canadian health policy & planning

Four broad implications of our results for health policy and planning seem most apparent. First, it would be useful to come to a greater understanding of what unattached patients’ expectations are of having a regular family doctor in order to determine whether or not they are realistic, and if they are not, whether this may be contributing to their lack of success in gaining attachment. Doing so is directly relevant to the success of attachment initiatives. Second, participants clearly favoured a ‘traditional’ model of being enlisted by a single family doctor with whom they can develop a long-term trusting relationship. Meanwhile, recent Canadian health reforms have worked to develop team or collaborative practice models [57,58]. Consideration needs to be given to how the benefits of such a practice style are conveyed to unattached patients so that their expectations are in line with the types of practices they might be enlisted into. Third, the role of physician choice in enlisting new patients needs to be carefully scrutinized, as was recently done in the province of Ontario [20], in order to determine whether or not this is resulting in the continued unattachment of certain patient populations given participants’ concerns that they were thought of as undesirable and thus not enlisted into practices. Forth, serious consideration needs to be given to the roles walk-in clinics should play in ongoing chronic illness management. If health care administrators view these clinics as having a role in such management then measures need to be put into place to enable doctors in these clinics to do things such as routine testing and remind patients about such tests.

The *Attachment Initiative* program in BC is still in its early stages. One of its first initiatives, the *A GP for Me* program, which is designed to increase attachment rates among high-needs patient populations such as people managing chronic illness, is not expected to be in place across the province until 2015 [59]. The *Attachment Initiative* is aiming to have communities develop community-based solutions to the problem of patient unattachment [17]. A number of implications or directions for the *Attachment Initiative* emerge from this analysis. First, it could be very useful to create a mechanism for tracking patients’ unsuccessful attempts at becoming enlisted in order to identify and thus respond to trends, including among chronically ill individuals. This could involve having physicians who are accepting patients report very basic information on who was or was not taken into their practice. Second, based upon the participants’ discussions of why they became unattached, it can be understood that there is a need to have information on doctors accepting new patients available in places where people in transitional life stages are located in addition to walk-in clinics. Third, acknowledging that having a 100% attachment rate in BC is a very long-term goal, interim solutions to the problems associated with managing a chronic illness without having a regular family doctor, such as obtaining referrals for needed blood work and minimizing repeated testing due to record unavailability, need to be developed and implemented as part of the *Attachment Initiative* program, with particular attention being paid to the role of walk-in clinic physicians.

### Limitations

There are three main limitations of this research. First, a limitation focus groups is that some participants may conform to the opinions of the group in ways that do not truly reflect their personal opinions [60]. In running the groups we saw no evidence of this. Second, we relied on participants’ self-reports that they were unattached and managing a chronic illness. While we have no reason to believe otherwise, this could nevertheless be the case. Third, our findings are not generalizable as we cannot determine how representative our sample is of all unattached patients in BC as such population-level data do not exist. The findings also represent the experiences of a group of marginalized patients who self-identified as unattached to a family doctor that: were fairly young relative to those who typically manage (multiple) chronic illness; had low socio-economic status; were heavily reliant on government assistance; resided in a single BC community; and had a significant history of drug use. The findings, though not ever intended to be generalizable because of the qualitative design [61], must always be considered in this light.

### Strengths

A significant strength of this study is that it gives voice to a group of marginalized individuals who are rarely heard from: unattached patients managing chronic illnesses. Although these patients are often missing from existing health services research, Canadian or otherwise, no doubt due in part to the challenges with identifying them, the findings show that there is much to be learned from their experiences. For example, the focus group discussions brought to the fore not only the challenges participants face but the hopes they have for obtaining attachment in the future, while providing very specific narrative examples of each. Such findings are reflective of the deep insights that can be generated from qualitative health research to inform policy and practice, particularly when coupled with quantitative data that can emphasize trends and gaps [36].

## Conclusions

This study has examined experiences of being unattached to a family doctor by 26 chronically ill individuals living in a low socio-economic status neighbourhood of a mid-sized BC city. Participants identified life events as the main reason for not having regular family doctor. They clearly perceived that there are many benefits to be had by having a regular family doctor. In particular, these unattached patients valued the trusting relationship that could be attained with a regular family doctor, and the knowledge a family doctor would have about their own behaviour regarding needs for prescriptions and paperwork completion for benefits. Participants also reflected on the sometimes harsh realities of not having a regular family doctor, including experiencing a lack of continuity in their medical records and a general inability to seek medical care in the face of symptom exacerbations. They were concerned that such realities could compromise their health given that they were managing chronic illnesses. We encourage more research to be done on the lived experience of unattachment in order to provide the on-the-ground insights that policy-makers and health care administrators require to develop responsive, patient-centred supports and programs.

## Abbreviations

BC, British Columbia.

## Competing interests

The authors declare that they have no competing interest.

## Authors’ contributions

VAC ran the focus groups, led the data analysis, and led the drafting of this article. GA was involved in conceptualizing the study, provided feedback on the focus group guide, confirmed themes and sub-themes, provided feedback on the article, and drafted one section and one sub-section of the article. AH reviewed the transcripts, coded data extracts by sub-theme, provided input into the analysis, and offered feedback on the article. All authors read and approved the final manuscript.

## Pre-publication history

The pre-publication history for this paper can be accessed here:

http://www.biomedcentral.com/1471-2296/13/69/prepub
